# A Review of the Pharmacological Effects of the Dried Root of *Polygonum cuspidatum* (Hu Zhang) and Its Constituents

**DOI:** 10.1155/2013/208349

**Published:** 2013-09-30

**Authors:** Huan Zhang, Chang Li, Sin-Tung Kwok, Qing-Wen Zhang, Shun-Wan Chan

**Affiliations:** ^1^Department of Applied Biology and Chemical Technology, The Hong Kong Polytechnic University, Hong Kong, China; ^2^Food Safety and Technology Research Centre, Department of Applied Biology and Chemical Technology, The Hong Kong Polytechnic University, Hong Kong, China; ^3^Industrial Liaison Office, Technological and Higher Education Institute of Hong Kong, Hong Kong, China; ^4^Institute of Chinese Medical Sciences, University of Macau, Macau, China; ^5^State Key Laboratory of Chinese Medicine and Molecular Pharmacology, Department of Applied Biology and Chemical Technology, The Hong Kong Polytechnic University, Hong Kong, China

## Abstract

Traditional Chinese medicine (TCM) has been widely used in China for thousands of years to treat and prevent diseases. TCM has been proven safe and effective, and it is being considered as one of the important types of complementary and alternative medicine and receives increasing attention worldwide. The dried root of *Polygonum cuspidatum* Sieb. et Zucc. (also known as “Hu Zhang” in Chinese) is one of the medicinal herbs listed in the Pharmacopoeia of the People's Republic of China. Hu Zhang is widely distributed in the world. It can be found in Asia and North America and is used as folk medicine in countries such as Japan and Korea. In China, Hu Zhang is usually used in combination with other TCM herbs. The therapeutic uses of those Hu Zhang-containing TCM prescriptions or formulations are for treating cough, hepatitis, jaundice, amenorrhea, leucorrhea, arthralgia, burns and snake bites. Recent pharmacological and clinical studies have indicated that Hu Zhang has antiviral, antimicrobial, anti-inflammatory, neuroprotective, and cardioprotective functions. This review gives a summary of the reported therapeutic effects of the active compounds and the different extracts of Hu Zhang.

## 1. Introduction

The definition of complementary and alternative medicine (CAM) is broad. In general, CAM refers to a group of health care systems, practices, and medications that are not considered conventional or orthodox. CAM includes traditional Chinese medicine (TCM), acupuncture, Ayurveda, massage therapies, and mind-body therapies (such as yoga). It is often used together with conventional medicine. It is common that patients with chronic diseases turn to CAM therapies for better treatment effects, fewer side effects, or for relieving side effects of drugs. TCM, a well-known CAM, has been used to treat a variety of diseases for thousands of years [1–3]. *Panax ginseng*, *Pinella ternate*, *Salviae miltiorrhizae *and *Arisaema japonicum* are some commonly known TCMs [1, 4, 5].

As one of the important types of CAM, TCM is receiving increasing attention among scientists worldwide. For treating some complex diseases such as diabetes mellitus and cancer, TCM is one of the common alternatives of conventional medications. In recent decades, researchers from mainland China, Hong Kong, and Taiwan have focused on the investigation of various TCM herbs and their active compounds and have discovered therapeutics that are based on single compounds, such as salvicine for anticancer activity and artemisinin for malaria treatment [6]. 

**Figure 1:**
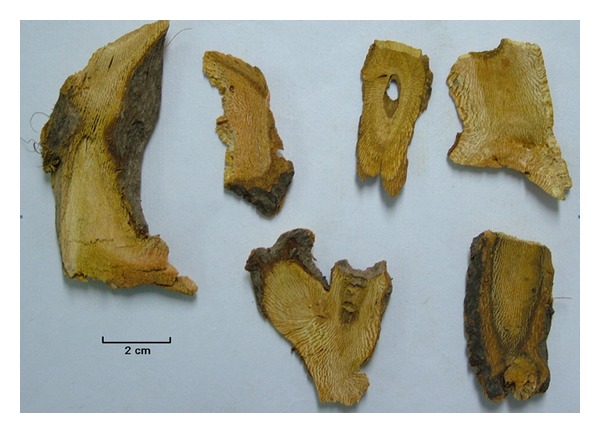
Photo of the dried root of *Polygonum cuspidatum* (Hu Zhang).

*Polygonum cuspidatum* Sieb. et Zucc. is a herbaceous perennial plant. It is a member of the genus *Polygonum* in the family Polygonaceae, which grows in Asia and North America. In China, there are about 80 species of *Polygonum* used in TCM [7]. Its dried root (Figure 1) is officially listed in the Pharmacopoeia of the People's Republic of China under the name “Hu Zhang” [7]; it is also used as folk medicine in Japan and Korea. From the perspective of TCM theory, Hu Zhang is used to remove jaundice and clear heat-toxin so as to promote blood circulation, dispel stasis, expel wind and dampness, dissipate phlegm, and suppress cough. Therefore, Hu Zhang is commonly prescribed by TCM practitioners for the treatment of cough, hepatitis, jaundice, amenorrhea, leucorrhoea, arthralgia, hyperlipidemia scald and bruises, snake bites, and carbuncles [8].

Hu Zhang is frequently used as a hepatoprotective and cholagogic drug in TCM. Its effects on hypertension, hyperlipidemia, and cardiovascular and neurodegenerative diseases have also been intensively investigated, both experimentally and clinically.

The purpose of this review is to provide a comprehensive overview of the pharmacological effects of Hu Zhang and to attract the attention of more researchers towards its use as an alternative medicine in clinical settings. Additionally, the major chemical components of Hu Zhang are summarized.

## 2. Active Ingredients Found in Hu Zhang

Hu Zhang contains various classes of chemicals. Stilbenes including resveratrol, polydatin, and anthraquinones such as emodin and its glycoside are the major compounds in Hu Zhang. Hu Zhang also contains flavonoids such as quercetin and (+)-catechin. The major active ingredients isolated from this herb are emodin, physcion, emodin 8-*O-*β**-D-glucopyranoside, 2-methoxy-6-acetyl-7-methyljuglone, citreorosein, (+)-catechin, polydatin, and resveratrol (Table 1). Recently, some new compounds such as polygonins A and B were also isolated from Hu Zhang [9]. However, their pharmacological effects are not yet identified.

## 3. Pharmacological Activities

Hu Zhang has been used in many TCM formulas to treat various ailments. It is also used as folk medicine to promote general physical health. Pharmacological researches and clinical studies have indicated that Hu Zhang extract and its major compounds possess antivirus, antimicrobial, anti-inflammatory, neuroprotective, and cardioprotective activities (Tables 1 and 2).

### 3.1. Antiviral Activities

Chronic hepatitis B virus (HBV) infection remains one of the most challenging global health problems, with more than 350 million people infected and at risk of hepatic decompensation, cirrhosis, and hepatocellular carcinoma. Potent oral antiviral agents have been approved to treat hepatitis B since 1998. Therapy with interferon alpha and nucleosides or nucleotide analogues is effective to treat hepatitis B by suppressing virus replication, reducing hepatitis activity, and preventing disease progression [10]. Meanwhile, almost 50 million people worldwide are infected with human immunodeficiency virus (HIV). The number of HIV-positive people continues to increase at an alarming rate in China and some other Asian countries [11]. Although current anti-HBV/HIV drugs could improve the quality of life for those infected patients, emerging drug resistance has driven the need to search for new anti-HBV/HIV agents and targets. 

Many natural compounds that exhibit anti-HIV activity have been identified. These include alkaloids [12], flavonoids [13], and polyphenols [14]. 70% EtOH extract of Hu Zhang was demonstrated to have inhibitory function against HIV-1-induced syncytium formation in C8166 lymphocytes with a 50% effective concentration (EC_50_) of 13.94 ± 3.41 *μ*g/mL. Through bioactivity-guided fractionation of Hu Zhang, (*E*)-resveratrol, 5,7-dimethoxyphthalide, (+)-catechin, and emodin 8-*O-*β**-D-glucopyranoside were shown to exhibit fairly strong antiviral activity against HIV-1-induced cytopathic effects in C8166 lymphocytes at noncytotoxic concentrations. This provides evidence for the “heat-clearing and detoxifying” functions of Hu Zhang and its antiviral activities [15]. 

Researchers have explored the efficacy of Hu Zhang extracts against HBV in HepG_2_ 2.2.15 human hepatoblastoma cell line by quantitative real-time polymerase chain reaction in search of effective antiviral agents. The expressions of HBeAg and HBsAg were determined by enzyme-linked immunosorbent assay. Results indicated that ethanol extract of Hu Zhang could inhibit the production of HBV with an effective minimal dose of 10 *μ*g/mL. Both water and ethanol extracts of Hu Zhang significantly increased the expression of HBsAg, whereas a higher dose of water extract (30 *μ*g/mL) inhibited the expression of HBeAg. However, both extracts showed some degree of cytotoxicity to the host cells [16]. It is not known whether the anti-HBV effect and cytotoxicity are due to the same compound or active fraction of the extracts. Further chemical and biological analyses are required to purify the active component(s) in Hu Zhang. 

### 3.2. Antimicrobial Effects

Dental caries is a dental biofilm-related oral disease. Chlorhexidine and antibiotics, generally used as anti-biofilm agents, have shown undesirable side effects such as extrinsic staining and bacterial resistance. Hu Zhang is shown to be a promising alternative medicine for preventing dental caries [17]. It has been reported that a Hu Zhang fraction (called F1), that is mainly composed of physcion, emodin, and resveratrol, could enhance fluoride activity against *Streptococcus mutans* (*S. mutans*) virulence. F1 also showed inhibitory effects against F-ATPase activity and acid production of *S. mutans* in biofilms. Therefore, F1 may be useful for preventing oral diseases, particularly those related to dental biofilm [18].

*Vibrio vulnificus *(*V. vulnificus*)could cause fetus septicemia with mortality rate of more than 50% within a few days after infection [19]. Research has demonstrated that the ethanol extract of Hu Zhang and its active compound, emodin, possess significant protective effects against *V. vulnificus* cytotoxicity and infection. It was identified that ethanol extract of Hu Zhang and emodin could protect RAW 264.7 and Hela cells from *V. vulnificus*-induced cytotoxicity* in vitro*. They could also inhibit* V. vulnificus *growth and survival in seawater and heart infusion broth. Pretreatment of ethanol extract of Hu Zhang (200 mg/kg) or emodin (20 mg/kg) can protect 8-week-old CD-1 mice infected with* V. vulnificus in vivo *[20]. This further suggests the antimicrobial activity of Hu Zhang.

### 3.3. Anti-Inflammatory Effects

Inflammation could cause a variety of diseases such as autoimmune diseases [21], neurodegenerative diseases [22], cardiovascular diseases [23], or cancer [24]. Nonsteroidal anti-inflammatory drugs and cyclooxygenase-2 (COX-2) inhibitors are commonly used to treat diseases related to inflammation, but the adverse effects on the gastrointestinal and cardiovascular systems have limited their clinical applications. 

The pathogenesis of arthritis, hepatitis, and acute lung injury (ALI) are somehow related to inflammation [25–27]. Thus, inflammatory response plays an essential role in the progression of these diseases. Although many anti-inflammatory drugs are available clinically to treat arthritis, hepatitis, and ALI, their efficacy is limited and they always come with side effects. Researches that aim at identifying botanical drugs with little toxicity and good therapeutic performance have been increasing [3, 28]. Extensive studies have indicated that the extract of Hu Zhang or its major constituents have anti-inflammatory activities that may benefit patients with arthritis, hepatitis or ALI.

The anti-inflammatory effects of the ethyl acetate extract of Hu Zhang were investigated in Freund's complete adjuvant (FCA)-induced arthritis model and serotonin-induced paw edema model in Sprague-Dawley rats* in vivo.* The ethyl acetate extract of Hu Zhang at 100 and 200 mg/kg significantly suppressed serotonin-induced swelling since 12 min after serotonin treatment. Consistently, in the FCA-induced arthritis model, the ethyl acetate extract of Hu Zhang at 200 mg/kg significantly suppressed FCA-induced joint swelling within 3 days, whereas the ethyl acetate extract of Hu Zhang at 100 mg/kg showed similar suppression within 5 days. Furthermore, the extract effectively inhibited positive responses of c-reactive protein and rheumatoid factor when compared with the untreated control in the FCA-induced arthritis model. Taken together, these findings suggested that the ethyl acetate extract of Hu Zhang could be a potent agent for rheumatoid arthritis treatment [29]. 

Intravenous administration of lipopolysaccharide (LPS) could lead to activation of various inflammatory mediators such as phospholipase A_2_ (PLA_2_) in the ALI rat model* in vivo*. Polydatin, an active compound of Hu Zhang, could up-regulate Clara cell secretory protein (CCSP) to inhibit PLA_2_, which may be one of the crucial protection mechanisms of polydatin in LPS-induced ALI. For further investigation, the human bronchial epithelia cells transformed by the SV40 T-antigen were chosen as the model to study the effect of polydatin on CCSP *in vitro*. Polydatin can promote the expression of CCSP in normal and LPS-stimulated cells [30]. Additionally, polydatin could protect mice against carbon tetrachloride-induced liver injury through anti-inflammatory and antioxidative effects *in vivo*. These effects are achieved through suppressing levels of hepatic malondialdehyde (MDA), tumour necrosis factor-alpha (TNF-*α*), interleukin 1 beta (IL-1*β*), COX-2, inducible nitric oxide synthase (iNOS), and nuclear factor-kappaB (NF*κ*B) and enhancing levels of superoxide dismutase (SOD), glutathione (GSH), glutathione transferase (GST), catalase (CAT), glutathione peroxidase (GPx), and transforming growth factor-beta 1 (TGF-*β*_1_) in the liver tissue. Therefore, polydatin may help people cope with oxidative stress and inflammation-related liver damage [31].

Hu Zhang extract (standardized to contain 20% trans-resveratrol) demonstrated comprehensive suppressive effects on inflammatory and oxidative stress. These effects are achieved through decreasing levels of TNF-*α*, interleukin, intranuclear NF*κ*B binding, c-jun-N-terminal kinase 1 (JNK 1), phosphotyrosine phosphatase-1B (PTP-1B), as well as reactive oxygen species (ROS) generation in mononuclear cells [32].

Emodin, an active compound in Hu Zhang, was shown to inhibit the expression of inflammatory-associated genes including iNOS, TNF-*α*, interleukin-10, I*κ*B kinase (IKK)-alpha, and IKK-gamma and to inhibit the nuclear translocation of NF*κ*B on LPS-induced inflammatory responses in RAW 264.7 macrophages [33].

Citreorosein, an anthraquinone derivative isolated from Hu Zhang, inhibited COX-2-dependent prostaglandin D_2_ generation and COX-2 expression in mouse bone marrow-derived mast cells stimulated with stem cell factor. The effect of citreorosein was achieved through inhibition of the Akt and JNK pathways [34].

Ethanolic solution of Hu Zhang and resveratrol were demonstrated to inhibit the development of edema and leukocyte infiltration in the 12-*O-*tetradecanoylphorbol-13-acetate-(TPA-) induced ear edema in mice* in vivo* [35]. Topical application of resveratrol also significantly inhibited TPA-induced COX-2 expression via modulation of the IKK-NF*κ*B signaling cascade in mouse skin *in vivo*. This investigation provides evidence for the potential uses of Hu Zhang in cosmeceutical and dermatological products [36]. 

### 3.4. Neuroprotective Activities

Preventing neuronal death is a top priority for treating neurological diseases [37]. Oxidative stress is implicated as a causative factor in neuronal death in neurodegenerative disorders [38]. There is a growing interest in searching for neuroprotective agents from natural products since they contain compounds with high antioxidant power [39]. Several studies have reported the neuroprotective effects of Hu Zhang extract or its major compounds such as polydatin, emodin 8-*O-*β**-D-glucopyranoside, 2-methoxy-6-acetyl-7-methyljuglone, and resveratrol.

It has been found that polydatin could reduce the volume of cerebral infraction and improve rat neurological deficits induced by transient middle cerebral artery occlusion (MCAO). Polydatin also protects the brain from injury by inhibiting the expression of cell adhesion molecules, in particular vascular cell adhesion molecule 1 (VCAM-1), intracellular adhesion molecule 1 (ICAM-1), L-selectin, and E-selectin. These findings suggest that polydatin may be a potential agent for treatment of brain injury associated with stroke [40]. Additionally, polydatin could markedly attenuate cognitive deficits induced by chronic cerebral hypoperfusion in rats, decrease the production of MDA and increase the activities of SOD and CAT. Additionally, polydatin has also exerted the protective effects in oxygen glucose deprivation (OGD) model. These results demonstrate that polydatin could offer a novel therapeutic strategy for the treatment of vascular dementia [41]. Apart from polydatin, emodin 8-*O-*β**-D-glucopyranoside (an anthraquinone) has been suggested to have protective effects against cerebral ischemia-reperfused injury *in vivo* and glutamate-induced damage in cortical cells* in vitro*. It decreased MDA level in the brain and increased SOD activity. Moreover, emodin 8-*O-*β**-D-glucopyranoside reduced the neurological deficit score and the cerebral infraction area [42]. Therefore, one of the important pathways for Hu Zhang to elicit its neuroprotective effects may relate to its antioxidant properties.

2-Methoxy-6-acetyl-7-methyljuglone, another anthraquinone isolated from Hu Zhang, could effectively protect PC12 cells against cytotoxicity induced by tert-Butyl hydroperoxide. The neuroprotective effect of 2-methoxy-6-acetyl-7-methyljuglone may contribute to its antioxidant effect and ability to decrease the expressions of the phosphorylation of ERK1/2, JNK, and p38 MAPK [43].

Senescence-accelerated mouse (SAM), an aging model, was used for brain aging and anti-aging pharmacology studies. Resveratrol extracted from Hu Zhang increased the SOD and GPx activities, while decreasing MDA level in SAM *in vivo*. Resveratrol could improve neuromuscular coordination and sensorimotor ability in tightrope test. It could also enhance the learning and memory capacity in Morris water maze test in SAM. These results indicate that resveratrol may exhibit therapeutic potential for age-related conditions [44].

### 3.5. Cardioprotective Activities

Hyperlipidemia is one of the major risk factors of cardiovascular diseases such as coronary heart disease and atherosclerosis. Natural products have been shown to be effective in modulating serum lipid profile under hyperlipidemic [4] or hypercholesterolemic [45, 46] conditions. Polydatin could markedly reduce the serum levels of triglycerides, total cholesterol, and low-density lipoprotein cholesterol in hyperlipidemic rabbits [47]. For the prominent beneficial effect on serum lipid profile, it is worth exploring polydatin as a hypidolipemic drug or health supplement for patients with hyperlipidemia and/or hypercholesterolemia.

Cardiomyocytes are sensitive to ischemia/reperfusion (I/R). Polydatin intravenously administrated strongly protects the myocardium against I/R injury by activating protein kinase C (PKC) and opening mitochondrial ATP-sensitive K^+^ channel. Meanwhile, pretreatment of polydatin-attenuated changes in MDA and SOD, suggests that polydatin might protect myocardial against I/R injury through free radical-elimination mechanism. The findings demonstrated that polydatin may have therapeutic potential in the treatment of cardiac reperfusion injury and other cardiovascular diseases that are related to mitochondrial oxidative damage in etiology [48]. Polydatin also has beneficial effects in ventricular remodeling induced by isoproterenol in mice and by abdominal aortic banding in rats *in vivo*. Its pharmacological effects on the heart are at least in part mediated by inhibiting the activation of renin-angiotensin-aldosterone system (RAAS) and decreasing the excretion of endothelin 1, TNF-*α*, and angiotensin II. Therapeutic use of polydatin might have potential in early treatment of chronic heart failure and improvement of ventricular remodeling [49].

Resveratrol could also protect the heart from I/R injury, prevent cardiac hypertrophy in hypertensive animals, and reduce the progression of atherosclerosis. It is believed that endothelial NOS, estrogen receptor alpha (ER*α*), Akt kinase, NF*κ*B, and survival activating factor enhancement pathway may mediate the aforementioned cardiovascular effects of resveratrol [50]. Resveratrol supplementation elevated apo-AI/apo B ratio and levels of HDL-cholesterol, and decreased plasma LDL-C concentration and hepatic HMG-CoA reductase activity. Moreover, in resveratrol-supplemented apo E^−/−^ mice, ICAM-1 and VCAM-1 in atherosclerotic vessels were diminished, thereby delaying the progression of atherosclerosis [51]. Pretreated with resveratrol improved cardiac function and reducement myocardial infarct size and cardiomyocyte apoptosis in the ischemic/reperfused rats heart. Resveratrol protected the ischemic heart by restoring the IR-induced altered microRNA expressions [52]. All of the above findings suggest that polydatin and resveratrol are potential bioactive compounds for treating cardiovascular diseases.

### 3.6. Other Activities

In recent years, resveratrol has become widely appreciated in the field of botanical dietary supplements [56]. Resveratrol, a dietary phenolic compound, in fruits and medicinal plants, exerts chemopreventive and antitumor effects [57]. Administration of resveratrol was found to protect salivary glands against radiation-induced dysfunction in mice. It can reverse the reduction of saliva secretion and restore salivary amylase and SOD activity. Resveratrol has great potential as a treatment for successful radiotherapy in clinical practice [58]. Resveratrol impeded cancer stem cells' (CSCs) properties through the activation of p53. Furthermore, resveratrol suppressed the stemness and epithelial-mesenchymal transition (EMT) through reactivating p53 and inducing miR-145 and miR-200c [59]. In MCF-7 breast cancer cells, resveratrol with doxorubicin can inhibit HSP expression and improve the therapeutic effects of doxorubicin probably by means of cell death induction. These findings suggest that resveratrol may be an effective adjuvant in breast cancer therapy [60]. 

 In addition, Hu Zhang's methanolic extract and active compounds, such as emodin and emodin 8-*O-*β**-D-glucopyranoside, were found to enhance the proliferation of MCF-7, an estrogen-sensitive cell line, in a concentration-dependent manner. It was found that emodin exerts estrogen-like activities by binding to human ER*α* and ER*β*. It may be useful for hormone replacement therapy against human menoxenia and post-menopausal diseases [53]. Citreorosein, a naturally occurring anthraquinone derivative from Hu Zhang, was found to exert estrogenic activity by using a recombinant assay [55].

Three Hu Zhang's anthraquinones, physcion, emodin, and citreorosein, showed moderate to strong tyrosinase inhibition. Thus, they may be used as skin whitening agents in place of kojic acid. Among these anthraquinones, physcion exhibited the most potent tyrosinase inhibition and showed higher permeability into the skin [54], suggesting its potential in cosmeceutical and dermatological uses.

## 4. Conclusions

Conventional medicines provide significant therapeutic benefits, but they also have side effects and they may have problem of drug resistance when same drugs are used over a long period of time. The search for TCM with fewer side effects and little toxicity has gained momentum over the years. The use of TCM has a long history with proven effectiveness and safety. Hu Zhang has been prescribed in China for medical purposes for thousands of years. Reports in the literature have demonstrated Hu Zhang's potential beneficial effects such as antimicrobial, antiviral, anti-inflammatory, estrogenic, neuroprotective, and cardioprotective activities. Yet, there is no research reporting/investigating the toxicity of Hu Zhang. Based on the results from both clinical tests and research tests conducted in laboratories, several active compounds of Hu Zhang have demonstrated positive effects on a variety of diseases. Hu Zhang might be a valuable alternative medicine that could be integrated into conventional treatments. More researches on the beneficial effects of Hu Zhang and its potential risks as an alternative medicine are needed.

## Figures and Tables

**Table 1:** Major active compounds of Hu Zhang and their pharmacological effects reported in the literature.

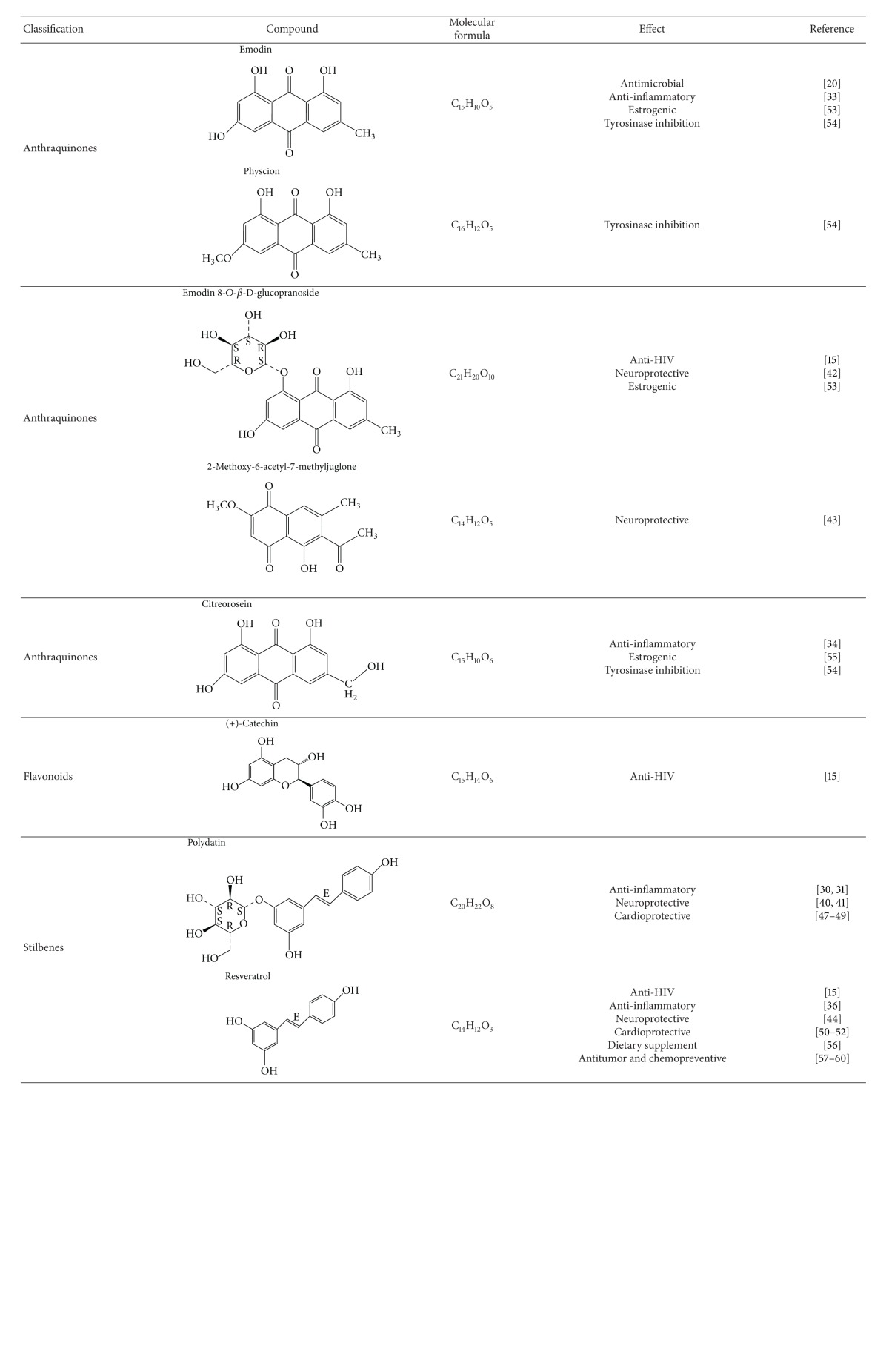

**Table 2:** A summary of Hu Zhang's and its major constituents' pharmacological activities.

Types of pharmacological activities	Types of active extract or compounds	Types of experiments	Testing subjects Administration route	Description of the effects	Reference
Antiviral activities	Ethanol extract Resveratrol, (+)-catechin Emodin 8-*O-*β**-D-glucopyranoside	*In vitro *	C8166 lymphocytes	Inhibit HIV-1-induced syncytium formation	[15]
	Water and ethanol extract	*In vitro *	HepG_2_ 2.2.15 cells	Inhibit the production of HBV Increase the expression of HBsAg Inhibit the expression of HBeAg	[16]

Antimicrobial effects	Hu zhang fraction F1	*In vitro *	*Streptococcus mutans *	Enhance fluoride activity against *S.mutans* virulence Inhibit F-ATPase activity and acid production of *S.mutans* in biofilms	[18]
	Ethanol extract Emodin	*In vitro *	RAW 264.7 Hela cells	Protect RAW 264.7 and Hela cells from *V. vulnificus*-induced cytotoxicity Inhibit* V. vulnificus *growth and survival in seawater and heart infusion broth	[20]
		*In vivo *	CD-1 mice Intraperitoneal route	Protect 8-week-old CD-1 mice infected with* V. vulnificus *	

Anti-inflammatory effects	Ethyl acetate extract	*In vivo *	Sprague-Dawley rats Orally administered	Suppress serotonin-induced swelling Suppress FCA-induced joint swelling Inhibit positive responses of c-reactive protein and rheumatoid factor	[29]
	Polydatin	*In vivo *	Male Sprague-Dawley Rats Intraperitoneal route	Upregulate CCSP to inhibit PLA_2_	[30]
		*In vitro *	BEAS-2B cells	Promote the expression of CCSP in normal and LPS-stimulated cells	
	Polydatin	*In vivo *	Male ICR mice Orally administered	Suppress levels of MDA, TNF-*α*, IL-1*β*, COX-2, iNOS, and NF*κ*B Enhance levels of SOD, GSH, GST, CAT, GPx, and TGF-*β*_1_	[31]
	Hu zhang extract	*In vitro *	Mononuclear cells	Decrease levels of TNF-*α*, IL-6, intranuclear NF*κ*B binding, JNK-1, PTP-1B, and ROS generation	[32]

Anti-inflammatory effects	Emodin	*In vitro *	RAW 264.7 macrophages	Inhibit the expression of iNOS, TNF-*α*, interleukin 10, IKK-alpha, IKK-gamma, and the nuclear translocation of NF*κ*B	[33]
	Citreorosein	*In vitro *	Mouse bone marrow-derived mast cells	Inhibit COX-2-dependent prostaglandin D_2_ generation and COX-2 expression through inhibition of the Akt and JNK pathways	[34]
	Ethanolic extract of resveratrol	*In vivo *	Female Swiss Webster mice Injectd in the inner and outer ear surfaces	Inhibit the development of edema and leukocyte infiltration	[35]
	Resveratrol	*In vivo *	Female ICR mice Topically to the dorsal shaven area	Inhibit TPA-induced COX-2 expression via modulation of the IKK-NF*κ*B signaling cascade in mouse skin	[36]

Neuroprotective activities	Polydatin	*In vivo *	Male Sprague–Dawley rats Sublingual vena injection	Reduce the volume of cerebral infraction Improve rat neurological deficits Protect the brain from injury by inhibiting the expression ICAM-1,VCAM-1, L-selectin, and E-selectin	[40]
	Polydatin	*In vivo *	Male Sprague-Dawley rats Orally administered	Attenuate cognitive deficits induced by chronic cerebral hypoperfusion in rats Decrease the production of MDA Increase the activities of SOD and CAT	[41]
		*In vitro *	Rat's primary cortical neurons	Alleviate the injuries of primary cortical neurons induced by OGD	
	Emodin 8-*O-*β**-D-glucopyranoside	*In vivo *	Male Wistar rats Tail vein injection	Reduce the neurological deficit score and the cerebral infraction area Decrease MDA level in the brain Increase SOD activity	[42]
		*In vitro *	Primary cortical cells	Decrease LDH release Increase mitochondrial activity	

Neuroprotective activities	2-Methoxy-6-acetyl-7-methyljuglone	*In vitro *	PC12 cells	Enhance antioxidative effect Decrease the expressions of the phosphorylation of ERK1/2, JNK, and p38 MAPK	[43]
	Resveratrol	*In vivo *	Male Sprague-Dawley rats Intragastric gavage	Increase the SOD and GPx activities Decrease MDA level Improve neuromuscular coordination and sensorimotor ability in tightrope test Enhance the learning and memory capacity in Morris water maze test in SAM	[44]

Cardioprotective activities	Polydatin	*In vivo *	Female Japanese Giant Ear Rabbits Orally administrated	Reduce the serum levels of triglycerides, total cholesterol, and LDL	[47]
	Polydatin	*In vivo *	Male Sprague-Dawley rats Intravenously administrated	Activate PKC and open mitochondrial ATP-sensitive K^+ ^channel Attenuate changes in MDA and SOD	[48]

Cardioprotective activities	Polydatin	*In vivo *	Male Kunming mice Male Sprague-Dawley rats Intragastrically administrated	Inhibit the activation of RAAS and decrease the excretion of endothelin-1, TNF-*α*, and angiotensin II	[49]
	Resveratrol	*In vitro *	Human cardiac AC16 cells	Activate STAT3 signaling Induce the expression of Bcl-xL	[50]
	Resveratrol	*In vivo *	Male apo E^−/−^ mice semisynthetic diet	Elevate apo-AI/apo B ratio and levels of HDL-cholesterol Decrease plasma LDL-C concentration and hepatic HMG-CoA reductase activity, ICAM-1, and VCAM-1 expression	[51]
	Resveratrol	*In vivo *	Male Sprague–Dawley rats Gavage administered	Improve cardiac function and reduce myocardial infarct size and cardiomyocyte apoptosis in the IR rat heart Restore the IR-induced altered microRNA expressions	[52]

Radiotherapeutic effect	Resveratrol	*In vivo *	ICR mice Intraperitoneally administered	Protect salivary glands against radiation-induced dysfunction in mice Reverse the reduction of saliva secretion and restore salivary amylase and SOD activity	[58]

Chemopreventive and antitumor effects	Resveratrol	*In vitro *	Human NPC cell lines Cancer stem cells	Impede CSCs properties through the activation of p53 Suppress the stemness and EMT through reactivating p53 and inducing miR-145 and miR-200c	[59]
	Resveratrol	*In vitro *	MCF-7 breast cancer cells	Inhibit HSP expression Improve the therapeutic effects of doxorubicin	[60]

Estrogen-like activities	Emodin Emodin- 8-*O-*β**-D-glucopyranoside	*In vitro *	MCF-7 cells	Enhance the proliferation of MCF-7 Exert estrogen-like activities by binding to human ER*α* and ER*β*	[53]
	Citreorosein	*In vitro *	Recombinant yeast (*Saccharomyces cerevisiae* strain BJ3505)	Possess estrogenic activity	[55]

Cosmeceutical and dermatological effects	Physcion, Emodin Citreorosein	*In vitro *	Dorsal skin from pigs	Inhibit tyrosinase activity and exert higher permeability into the skin	[54]

## Abbreviations

HBV: Chronic hepatitis B virus,
TCM: Traditional Chinese medicine,
CAM: Complementary and alternative medicine,
HIV: Human immunodeficiency virus,
I/R: Ischemia/reperfusion,
ALI: Acute lung injury
FCA: Freund's complete adjuvant,
COX-2: Cyclooxygenase-2,
LPS: Lipopolysaccharide,
PLA_2_: Phospholipase A_2_,
MDA: Malondialdehyde,
TNF-*α*: Tumour necrosis factor-alpha,
IL-1*β*: Interleukin-1 beta,
iNOS: Inducible nitric oxide synthase,
NOS: Nitric oxide synthase,
NF*κ*B: Nuclear factor-kappaB,
SOD: Superoxide dismutase,
GSH: Glutathione,
GST: Glutathione transferase,
CAT: Catalase,
GPx: Glutathione peroxidase,
TGF-*β*_1_: Transforming growth factor-beta 1,
ER: Estrogen receptor,
JNK-1: c-jun-N-terminal kinase-1,
PTP-1B: Phosphotyrosine phosphatase-1B,
ROS: Reactive oxygen species,
IKK: I*κ*B kinase,
TPA: 12-*O*-tetradecanoylphorbol-13-acetate,
*S. mutans:*: * Streptococcus mutans,*
*V. vulnificus*: *Vibrio vulnificus,*
CCSP: Clara cell secretory protein,
MCAO: Middle cerebral artery occlusion,
VCAM-1: Vascular cell adhesion molecule-1,
ICAM-1: Intracellular adhesion molecule-1,
OGD: Oxygen-glucose deprivation,
PKC: Protein kinase C,
RAAS: Rennin-angiotensin-aldosterone system,
CSCs: Cancer stem cells,
EMT: Epithelial-mesenchymal transition.
